# Apparent diffusion coefficient and kurtosis parameters show early response to anti-angiogenic therapy in patients with colorectal liver metastases

**DOI:** 10.1186/s40644-026-01063-3

**Published:** 2026-06-04

**Authors:** Mihaela Rata, Khurum Khan, David J. Collins, Matthew R. Orton, James d’Arcy, Nina Tunariu, Maria Antonietta Bali, Ian Chau, Nicola Valeri, David Cunningham, Dow-Mu Koh

**Affiliations:** 1 MRI Unit, Department of Radiology, The Royal Marsden NHS Foundation Trust, Downs Road, London, SM2 5PT UK; 2 Division of Radiotherapy and Imaging, The Institute of Cancer Research, London, UK; 3 GI and Lymphoma Unit, Department of Medicine, The Royal Marsden NHS Trust, London and Sutton, UK; 4 Feinberg School of Medicine Northwestern University, University College London Hospital/UCL Cancer Institute, London, UK

**Keywords:** Diffusion weighted imaging, Kurtosis, Colorectal liver metastasis, Clinical trial

## Abstract

**Background:**

To investigate whether the diffusion kurtosis imaging (DKI) technique can identify tumour response to an anti-angiogenic therapy in a pilot study of patients with RAS-mutant colorectal liver metastases.

**Methods:**

This prospective imaging study enrolled 20 participants receiving Regorafenib treatment. A target metastasis > 2 cm in each patient was imaged before and at 15 days after treatment on a 1.5T MR scanner using a coronal, free-breathing DKI protocol. Data were motion-corrected and modelled using both the mono-exponential and DKI models. Median values derived from voxel-wise analysis of the whole delineated tumour were reported for three parameters [apparent diffusion coefficient (ADC, 10^− 3^ mm^2^/s), apparent kurtosis (K, a.u.) and kurtosis-corrected apparent diffusion (D, 10^− 3^ mm^2^/s)] before and after treatment. A 5-patient supplementary cohort was used to assess the repeatability of DKI parameters using the Bland-Altman analysis. Changes in pre- and post-treatment measurements of the three parameters were assessed using Wilcoxon signed-rank tests (*P* < 0.05 was considered significant). Diffusion weighted imaging (DWI) and DKI parameter correlations were evaluated with Spearman tests. Functional MR parameters were also compared against Response Evaluation Criteria In Solid Tumours v.1.1 (RECIST) evaluations.

**Results:**

Significant treatment-induced changes across the cohort were observed for all parameters: K decrease (0.907 vs. 0.786, *P* < 0.01; 13.3%), D increase (1.256 vs. 1.386 × 10^− 3^ mm^2^/s, *P* < 0.001; 10.4%) and ADC increase (0.910 vs. 1.035 × 10^− 3^ mm^2^/s, *P* < 0.001; 13.7%). For both visits, Spearman correlation tests found a moderate negative correlation between K and D, (*r*=-0.70; *P* < 0.001 and *r*=-0.58; *P* < 0.01) and a strong positive correlation (*r* = 0.84 and 0.88; P < < 0.001) between ADC and D. The repeatability R was lowest for K (51%), followed by D (12%), and ADC (9.4%). When compared to RECIST v.1.1 evaluations, K identified no clinical responders, whilst D and ADC identified 7/12 and 11/12 responders.

**Conclusions:**

Both ADC and DKI parameters showed a significant early cohort change to the anti-angiogenic effects of Regorafenib treatment in RAS-mutant colorectal liver metastases. The ADC parameter performed better than the K parameter in identifying patients benefitting from the treatment.

**Trial registration:**

NCT03010722 clinicaltrials.gov; registration date 6^th^ January 2015.

**Supplementary Information:**

The online version contains supplementary material available at 10.1186/s40644-026-01063-3.

## Background

The liver is the commonest site of metastatic disease for patients with colorectal cancer, with at least 25% patients developing colorectal liver metastases [1]. Usually, the response to chemotherapy in liver metastasis is based on size changes, as described by Response Evaluation Criteria In Solid Tumours (RECIST) v.1.1 [2]. Nevertheless, therapy may also change the tissue structure and cellular density, in addition to the tumour size, which can by investigated using functional imaging techniques such as diffusion-weighted imaging (DWI).

DWI is a valuable method of characterising tumour cellularity and assessing tumour response to therapy via the apparent diffusion coefficient (ADC) measurement [3]. DWI is based on the premise that the displacements of water within the imaged tissue due to diffusion have a gaussian distribution, whilst, in reality, there are deviations from this assumption due to the complexity of interactions between the intracellular and extracellular water in tissues. Hence, diffusion kurtosis imaging (DKI) has been proposed [4], which incorporates a non-gaussian distribution component to provide a more complex tissue diffusion model. The two outputs of the DKI model are the apparent kurtosis (K) and kurtosis-corrected apparent diffusion (D). These metrics have been found to be strongly linked to cellular microstructure and heterogeneity in tissues, as shown by several studies investigating DKI outside of the brain [5], including the head and neck [4], prostate [6, 7], breast [8, 9], lung [10] and liver [11–13], for both cancer diagnosis/grading and treatment response assessment.

A recent review [14] found 9 DKI studies for assessing hepatocellular carcinoma. Nevertheless, DKI use for assessing colorectal liver metastasis has been more limited, with only two papers reporting treatment response assessment [15, 16] and one reporting metastatic characterisation (RAS mutation detection) [17].

In this pilot study, we assess the tumour response to an anti-angiogenic therapy using DKI in a cohort of 20 patients with RAS-mutated colorectal liver metastases. The DKI results derive from a sub-cohort of a prospective single-centre phase II clinical trial assessing colorectal liver metastases (with a RAS mutation) treated with single-agent Regorafenib (Stivarga^®^). It is expected that the drug primarily impacts the tumour perfusion, which further results in extended necrosis and, indirectly, an increase in ADC that can be seen as a secondary effect. The full cohort report [18] demonstrated the clinical efficacy of the Regorafenib drug treatment and was mainly assessed by Dynamic Contrast Enhancement (DCE)-MRI (primary imaging endpoint) and clinical biomarkers. Our evaluation of the DKI technique (secondary imaging endpoint) aims to determine its suitability for clinical practice for assessing treatment response of colorectal liver metastases, without the need for exogenous contrast injection.

## Methods

### Study population

The trial recruitment was approved by our institutional review board (National Research Ethics Service, NRES Committee London-Fulham; Research Ethics Committee reference 14/LO/1812) and carried out in accordance with the Declaration of Helsinki. Informed written consent to participate to this prospective phase II study (NCT03010722 registry number on clinicaltrials.gov; registration date 6^th^ January 2015) was obtained from each patient.

The trial enrolled 25 patients with colorectal liver metastases (March 2015 to May 2016) for a single drug Regorafenib treatment (oral multi-kinase inhibitor with anti-angiogenic activity administered daily). The three trial inclusion criteria were: (1) patients at least 18-year-old with a WHO performance status of 0–1 for which all conventional treatments were exhausted; (2) patients had metastatic disease amenable to biopsy and repeat measurements with DCE-MRI; (3) patients were confirmed to have a RAS-mutant cancer type. All patients were scanned twice: before (day − 7 to 0) and at day 15 after treatment. At the end of the trial-specific imaging protocol, a further 5-min optional scanning using DKI was proposed to the patient; 22/25 patients consented to this additional scanning sequence. In addition, for this sub-cohort, an additional inclusion criterion was related to tumour size being > 2-cm diameter. The final DKI cohort study consisted of 20 patients, as two patients were further excluded due to their target metastasis being too small. Out of the 20 patients, 17 benefitted by disease control rate, measured by RECIST v.1.1, at 8 weeks post-treatment.

Subsequentially, five additional patients with liver metastases were recruited (February to June 2017) to assess same-day test-retest repeatability of the DKI technique. Note that these patients were not part of the original clinical trial.

### DKI acquisition

All imaging was performed on a 1.5T MR scanner (MAGNETOM Avanto, Siemens Healthcare, Erlangen, Germany), see full DKI parameters in Table 1. Free-breathing coronal DKI was acquired using a 2D echo planar imaging sequence with a 3-direction scan trace method and 5 b-values (range 100–1500 s/mm^2^). No b-values > 1500 s/mm^2^ were acquired, to ensure we are not reaching the noise floor. DWI for each gradient direction was obtained, and 4 independent acquisitions (no averaging) were acquired over 5 min. The same protocol was used for both trial and repeatability cohorts.

**Table 1:** MR parameters for the DKI sequence

DKI Parameters	Liver tumours (*n* = 20 patients)
	2D single shot echo planar imaging
Acquisition plane	Coronal
Breathing mode	Free breathing
Total acquisition time [min: s]	05:04
Time per single acquisition [min: s]	01:21
Number of individual acquisitions (NSA)	4
Acquired voxel size [mm^3^]	3.1 × 3.1 × 5.5
Reconstructed voxel size [mm^3^]	1.56 × 1.56 × 5
Slice thickness [mm]	5
TR [ms]	4500
TE [ms]	68
Slices per slab	20
Slice gap [mm]	0
Matrix (FE x PE)	128 × 128
FOV [mm^2^]	400 × 400
Receiver bandwidth [Hz/Pixel]	1954
Parallel acquisition (GRAPPA) (PE acceleration factor x reference lines)	2 × 30
Phase partial Fourier	7/8
Fat suppression	SPAIR
5 b-values [s/mm^2^]	100, 500, 1000, 1250, 1500
Diffusion times [ms]	δ = 14.6; Δ = 24

TR=repetition time; TE=echo time; FE=frequency encoding; PE=phase encoding; FOV=field of view; GRAPPA=GeneRalized Autocalibrating Partial Parallel Acquisition; SPAIR=SPectral Attenuated Inversion Recovery

### DKI processing

**Fig. 1:**
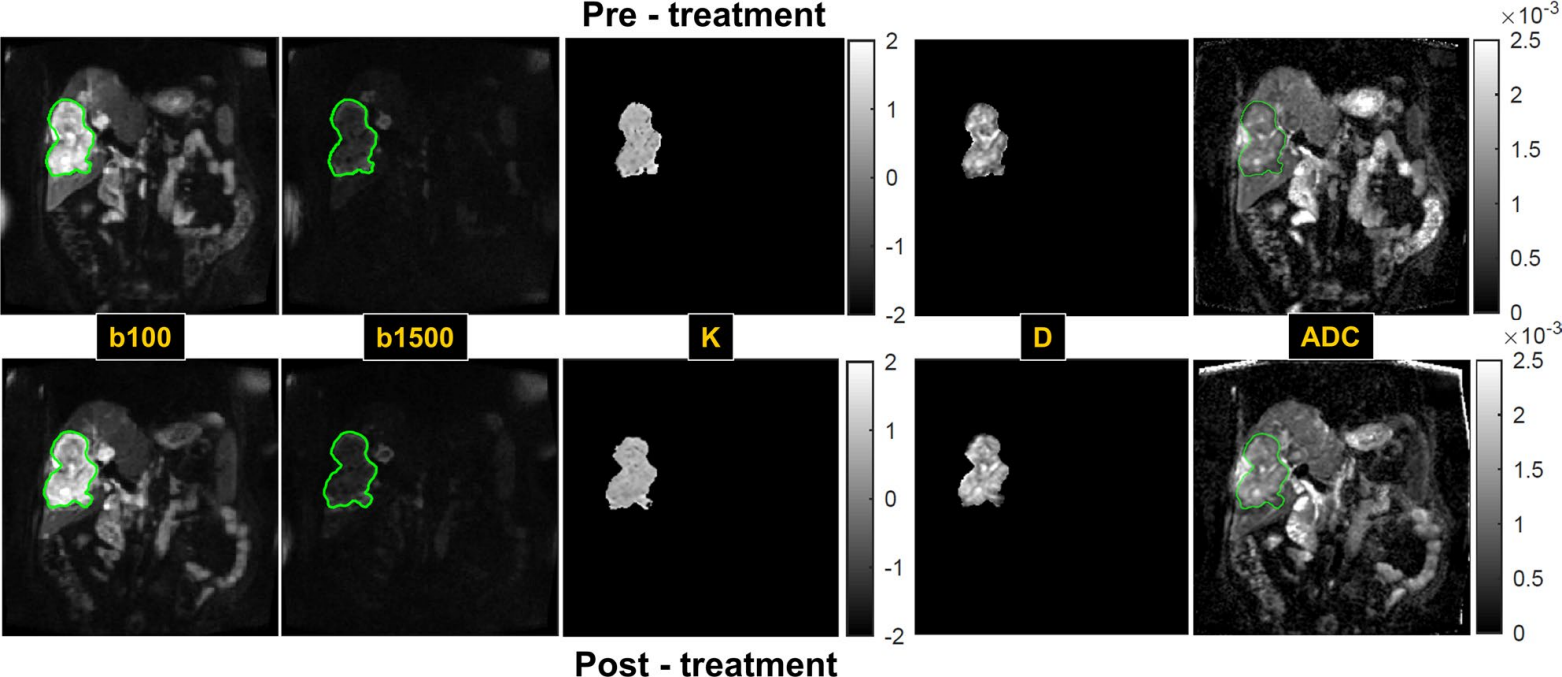
Example of K, D and ADC maps derived from a 53-year-old female patient with liver metastasis before (top row) and after 15 days of treatment (bottom row); main cohort; coronal plane; central slice showing a small necrotic core at the centre of lesion and a hyperintense tumour rim clearly visible on the high b-value image. Units are s/mm^2^ for the b100 and b1500 images, a.u. for K and mm^2^/s for D and ADC

**Fig. 2:**
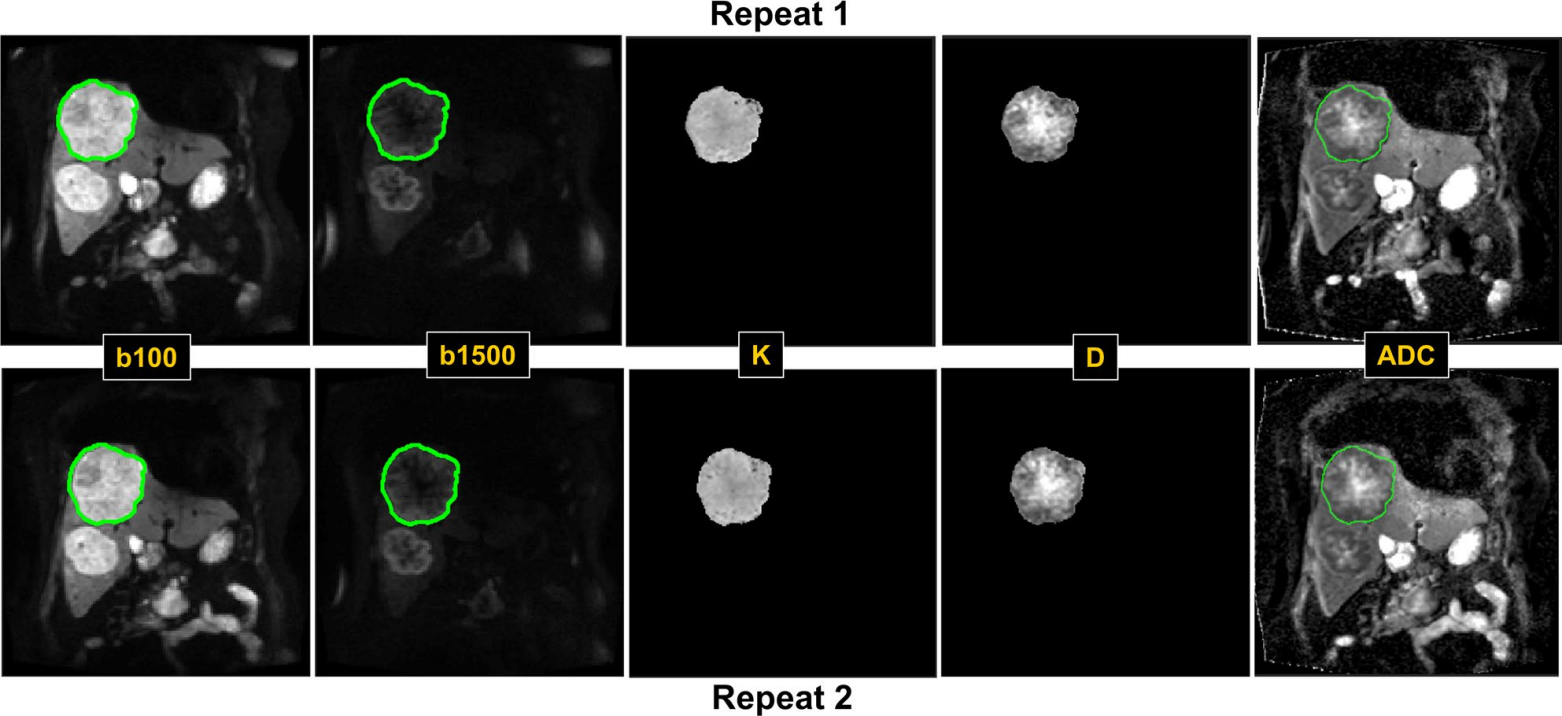
Similar example of K, D and ADC maps derived from a 50-year-old male patient with liver metastasis on two repeated scans obtained the same day; repeatability cohort; coronal plane; central slice showing a necrotic core at the centre of lesion. Units are s/mm^2^ for the b100 and b1500 images, a.u. for K and mm^2^/s for D and ADC

MRI data were motion-corrected (2D techniques, Matlab 2019a, see Supplementary material), and tumour volumes of interest (VOI) were drawn by a radiologist-supervised physicist on high-b-value images, at each visit for each patient of both cohorts (see Figs. 1 and 2). The VOI covered all tumour (regions of interest were drawn on each slice where tumour was visible) and the full heterogenous tumour (including necrotic cores) was considered. Voxel-wise analysis of the delineated VOI was performed for every patient, and median values of the parameters of interest were reported before/after treatment and for the two repeatability visits, respectively.

Two models using an in-house Matlab script (Matlab 2019a) were utilised to fit the data: 1). DKI model [4]; and 2). Mono-exponential diffusion model [19]. The three reported parameters were: apparent kurtosis (K) and kurtosis-corrected apparent diffusion (D) for the DKI model; and apparent diffusion coefficient (ADC) for the mono-exponential model. The following constraints were used for D (min = 1e^− 6^ and max = 5e^− 2^ mm^2^/s) and K (min = -1 and max = 10, a.u.) as part of the DKI model.

### Statistical analysis

Treatment-induced changes on all parameters were assessed with Wilcoxon signed-rank tests as parameters were not normally distributed. Correlations between K, D and ADC parameters were assessed by Spearman tests. The DKI repeatability was assessed with Bland-Altman analysis. For all analyses, a P-value of < 0.05 was deemed statistically significant.

## Results

### Patient demographics

The clinical characteristics of the 20 patients (7 women, 13 men; mean age 66.0 ± 9.9 years) that successfully completed the DKI study are presented in Table 2, together with another 5 patients (2 women, 3 men; mean age 61.0 ± 11.6 years) that underwent the additional short-term test-retest repeatability study.

**Table 2:** Clinical characteristics of patients for the main study and repeatability cohorts

Main cohort (*n* = 20): phase II clinical trial patients	
Disease	liver metastases from colorectal cancer
Primary cancer	colorectal (all patients)
Therapy	oral anti-angiogenic drug (Regorafenib) administered daily
Sex [Female/Male]	7/13
Age in years (mean ± SD; range)	66.0 ± 9.9; 52–86
Lesion volume in mL (mean ± SD; range)	51.7 ± 65.5; 2.7–265.1

**Repeatability cohort (n = 5): clinical patients**	
Disease	liver metastases from gastrointestinal cancer
Primary cancer	3 colorectal, 1 stomach, 1 caecal
Therapy	not relevant
Sex [Female/Male]	2/3
Age in years (mean ± SD, range)	61.0 ± 11.6; 50–80
Lesion volume in mL (mean ± SD, range)	91.9 ± 164.5; 4.1–385.6

SD=standard deviation

### DKI assessment of treatment response

**Fig. 3:**
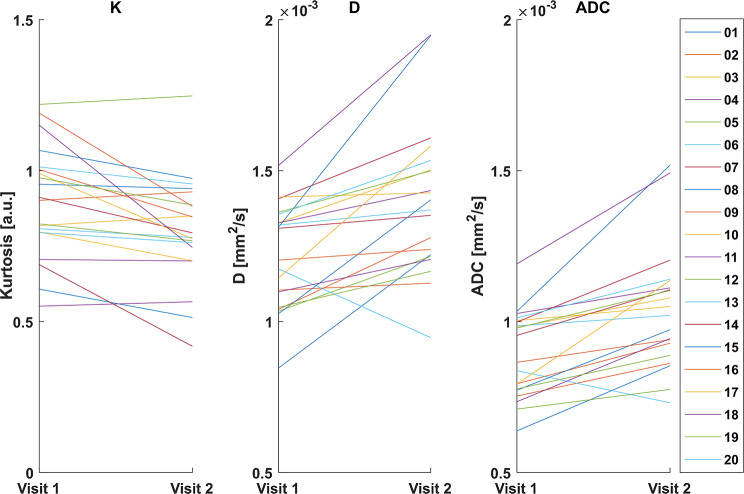
Pre/post-treatment ladder plots of K, D and ADC parameters from 20 liver metastases patients demonstrating significant cohort response for all parameters. Corresponding Wilcoxon P-values are listed in Table 3

**Fig. 4:**
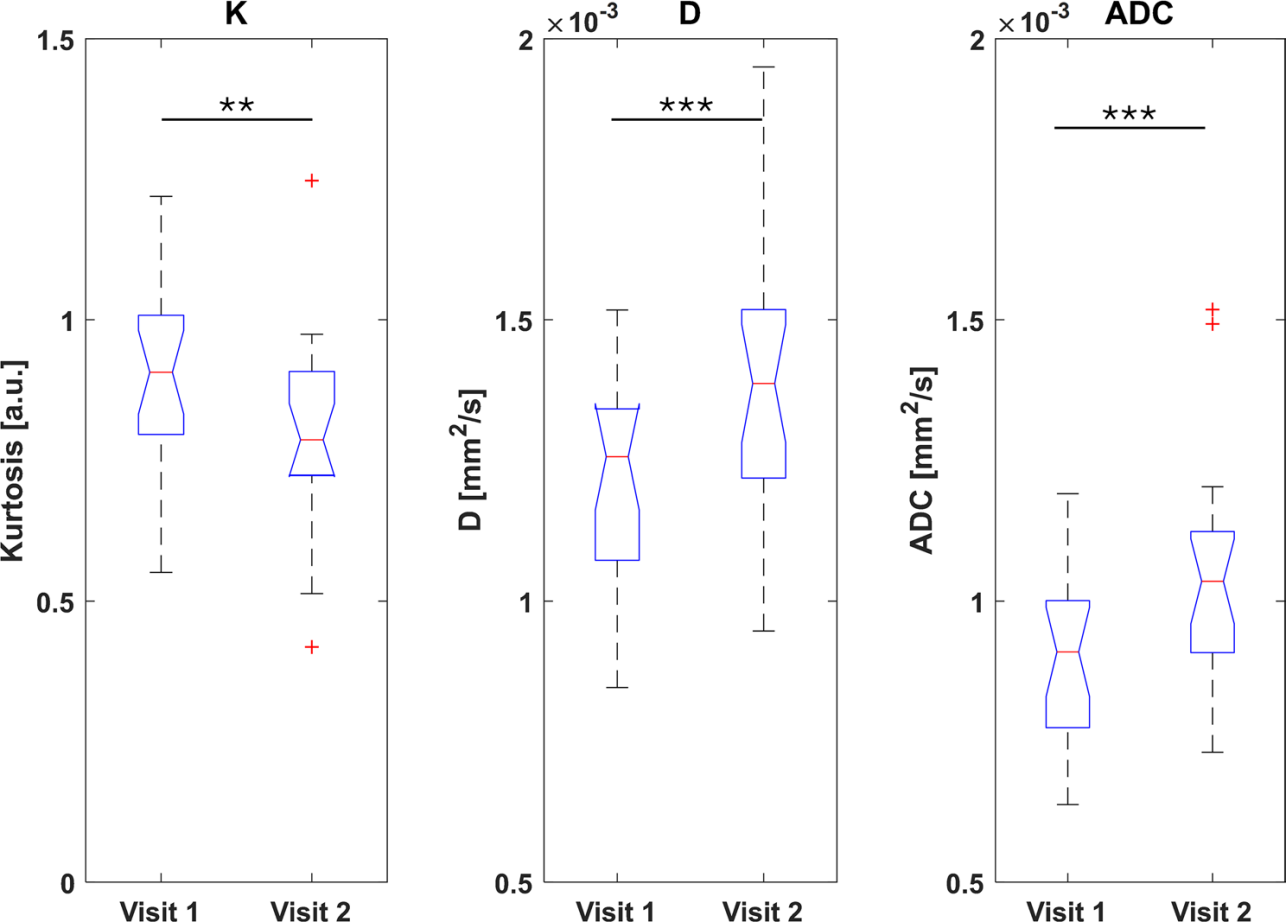
Pre/post-treatment boxplots of K, D and ADC parameters from 20 liver metastases patients demonstrating significant cohort response for all parameters. Corresponding Wilcoxon P-values are listed in Table 3

The treatment response of the DKI study cohort is shown in Fig. 3 (ladder plots of tumour median values of individual patients) and Fig. 4 (box plots). Statistically significant treatment-induced reduction was observed for K (0.907 vs. 0.786, *P* < 0.01; 13.3%) and increase for both D (1.256 vs. 1.386 × 10^− 3^ mm^2^/s, *P* < 0.001; 10.4%) and ADC (0.910 vs. 1.035 × 10^− 3^ mm^2^/s, *P* < 0.001; 13.7%) parameters. A typical example of MR parametric maps from a 53-year-old woman with liver metastasis is shown in Fig. 1, demonstrating a slight increase in D and ADC after treatment. All results are summarized in Table 3.

**Table 3:** Median values and main statistics of the DWI/DKI parameters (Wilcoxon test; paired t-test and Bland-Altman analysis)

Main cohort (*n* = 20)	Model: Kurtosis		Model: ADC
	K	D	ADC
	a.u.	x10^− 3^ mm^2^/s	x10^− 3^ mm^2^/s
Median value [Visit 1]	0.907	1.256	0.91
Median value [Visit 2]	0.786	1.387	1.035
P value (Wilcoxon signed-rank test)	0.0012	0.00068	0.00025
significance of statistical test	**	***	***
	*P* < 0.01	*P* < 0.001	*P* < 0.001

**Repeatability cohort (n = 5)**	**K**	**D**	**ADC**
	**a.u.**	**x10** ^**− 3**^ **mm** ^**2**^ **/s**	**x10** ^**− 3**^ **mm** ^**2**^ **/s**
Mean value [Repeat 1]	0.836	1.296	0.954
Mean value [Repeat 2]	0.599	1.372	0.951
P value (paired t-test)	0.39	0.80	0.60
*Bland-Altman tests*			
mean bias	-0.08	0.0114	0.0136
R [%]	51	12	9.4

### Correlation of DKI and ADC parameters

**Fig. 5:**
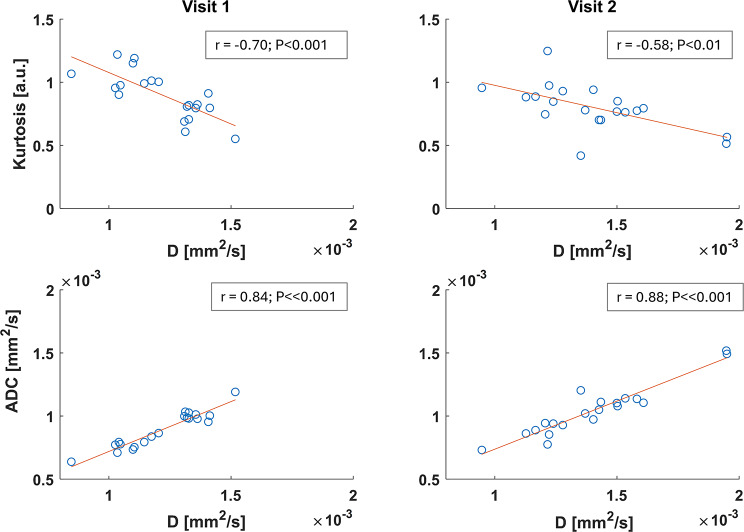
Scatter plots for K vs. D (top row) and ADC vs. D (bottom row) showing moderate correlation for K vs. D and strong correlation for ADC vs. D. Spearman’s rank correlation coefficient r and its corresponding P-value are shown. Pre- and post-treatment data from the main cohort of 20 patients with liver metastases

Scatter plots for K vs. D and ADC vs. D are presented in Fig. 5. Spearman correlation tests found a moderate negative correlation (0.5 < *r* < 0.7) between K and D, at both visits (*r*=-0.70; *P* < 0.001 and *r*=-0.58; *P* < 0.01), whilst ADC and D were strongly positive correlated (*r* = 0.84 and 0.88; P < < 0.001), at both visits, see Table 4.

**Table 4:** Spearman correlation of the DWI/DKI parameters at each visit and their statistics significance

Main cohort (*n* = 20)	K vs. D		ADC vs. D	
	Visit 1	Visit 2	Visit 1	Visit 2
Spearman coefficient r	-0.70	-0.58	0.84	0.88
P-value	0.00091	0.008	0	0
significance of statistical test	***	**	***	***
	*P* < 0.001	*P* < 0.01	P < < 0.001	P < < 0.001

### Repeatability of DKI parameters

**Fig. 6:**
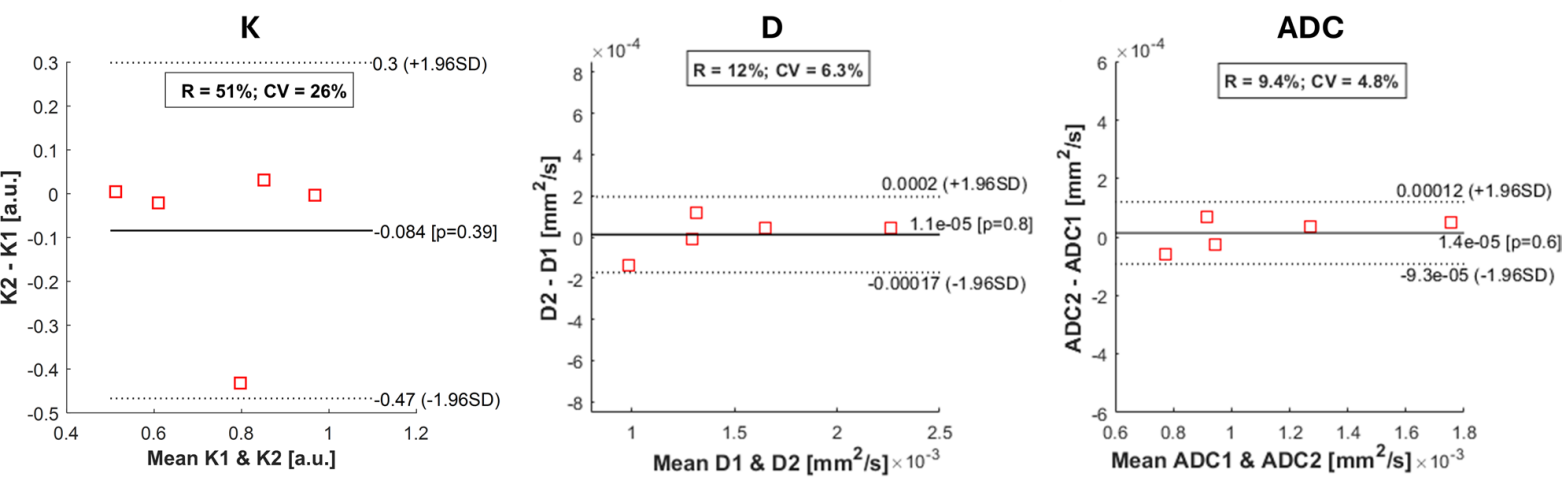
Bland-Altman plots for K, D and ADC parameters presenting values for the mean bias (and its P value), upper and lower limits of agreement and coefficient of variation and repeatability coefficient. Data derived from the 5-patient repeatability cohort

Individual Bland Altman plots for the three parameters (derived from the five-patient cohort) are presented in Fig. 6. The repeatability was worst for K (51%), followed by D (12%), and ADC (9.4%). Full statistical results are presented in Table 3. The mean bias was not statistically significant for any of the three parameters.

### Correlation of MR parameters and RECIST

**Fig. 7:**
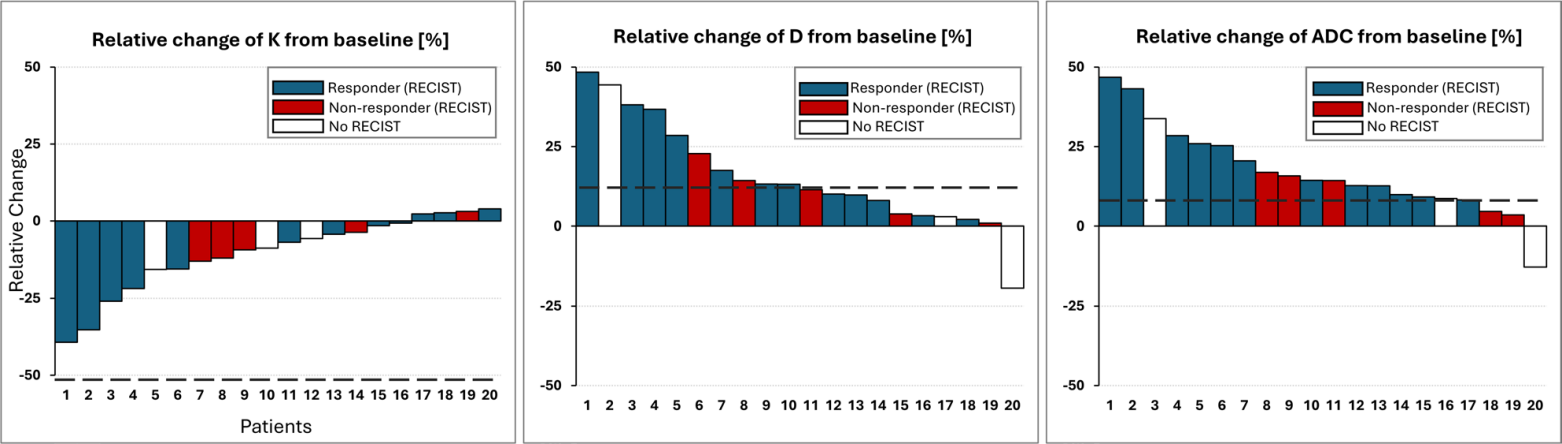
Waterfall plots for the K, D and ADC parameters demonstrating a good identification of responders. The percentage change (relative to baseline value) for each MR parameter was calculated at day 15 post-treatment (20 patients), while the RECIST was performed at week 8 (17 patients). The dotted line represents the limit of the repeatability (as measured from a 5-patient cohort) for each parameter

Figure 7 presents waterfall plots of the three MR parameters. Note that the disease control rate, measured by RECIST v.1.1, was obtained at a later timepoint (week 8 post-treatment) and covered 17/20 patients (three patients were not evaluated by RECIST), split into 12 patients who were considered to have benefited from treatment (responding or stable disease) and 5 who did not benefit (progressive disease). Whilst considering the repeatability R% threshold for the three MR parameters (as derived from a 5-patient cohort, and seen as a dotted line in Fig. 7), we found that, out of the 17 patients with RECIST v.1.1 evaluations available, K identified no patients with clinical benefit, while D and ADC correctly identified 7/12, and 11/12 respectively.

## Discussion

This 20-patient DKI/DWI study was a sub-cohort of a prospective single-centre trial assessing RAS-mutant colorectal liver metastases (with a specific genetic mutation) treated with single-agent Regorafenib, known for its anti-angiogenic and anti-proliferative effects. The main cohort results reported elsewhere demonstrated a significant treatment response as measured primarily by DCE-MRI parameters (also at 15 days weeks post treatment, i.e. at same time as the DKI/DWI assessments) that correlated, at later timepoints, with RECIST v.1.1 measurements [20] and standard clinical biomarkers [18].

This sub-cohort study found strong statistical significance of pre/post-treatment measurements for both DKI and DWI suggesting that kurtosis data may provide treatment response information as well. Moreover, the Spearman moderate negative correlation found between K and D, at both visits, may suggest a complementary sensitivity of these two DKI parameters. Conversely, a very strong correlation was found between D and ADC, suggesting that, in this cohort, the DKI model did not strongly impact the estimate of D. It is important to note that these early results were observed in the context of an anti-angiogenic drug trial, where the drug impact on DKI/DWI is expected to be relatively weak.

The main aim of this observational study was to investigate the usefulness of DKI/DWI parameters in describing early treatment response in a small cohort of RAS-mutant colorectal liver metastases. Due to the small cohort, response prediction was not directly assessed in this study, but the available RECIST information gave a better context in interpreting our findings.

The K parameter was considerably less repeatable (R%=51%) compared with D (12%) or ADC (9%), as suggested by measurements performed from a 5-patient cohort. Our results are somewhat different from a study [21] that reported, for hepatocellular cancer, a slightly better within coefficient of variation for K (8.79%, i.e. R%=24.35%) than for D (12.88%, i.e. R%=35.68%), as measured from an 18-patient cohort. The reported study [21] assessed respiratory-triggered axial DKI, whilst the present study explored free-breathing (with motion correction) coronal DKI, which may explain such difference.

With the tightest repeatability threshold (9%) among the three MR parameters, only the ADC showed a very good correlation with RECIST v.1.1 values. On the other hand, the large repeatability threshold for K (51%) implied that all treatment-induced changes measured for K were within the range of measurement error, and hence no correlation with RECIST was feasible. Note, however, that logistical limitations prevented us from acquiring a large enough cohort size for repeatability assessment. The very small sample of 5 patients was therefore aimed rather at suggesting an order of the R% values among the three parameters, rather than an explicit usefulness as R% threshold. We would expect that, with a bigger cohort size, the repeatability values should improve, which, in turn, might change the number of responders identified by each type of parameter. Unfortunately, the discrepancy of post treatment timepoints for DKI (2 weeks) and RECIST measurements (8 weeks) limits their direct comparison, as during those 6 weeks, a patient classification (responder or non-responder) has ample time to change one way or another.

Across the whole cohort, we observed a statistically significant post-treatment decrease of the baseline K and increase in D and ADC. The same pre/post-treatment trends for the three parameters were observed by Zhang et al. [16], although in their case the changes were not statistically significant. Only two recent studies assessed the use of K for predicting the chemotherapeutic response of colorectal liver metastases [15, 16]. Both studies demonstrated that the DKI model may predict early response to chemotherapy in patients with colorectal liver metastasis, albeit with some noted discrepancy. Zhow et al. [15] found baseline K was significantly lower in responding vs. non-responding lesions, whilst Zhang et al. [16] found the contrary, i.e. high baseline K associated with a good response to chemotherapy. Therefore, due to limited research and absence of method standardization, the current clinical usefulness of DKI remains uncertain. A recent review and meta-analysis of DWI [22] found that a lower baseline ADC (derived from mono-exponential model) was the most reliable predictor of response to treatment for colorectal liver metastases, better than non-mono-exponential models. Similarly, we found that the post-treatment increases of ADC and D were stronger statistically (*P* < 0.001) than the decrease in K (*P* < 0.01), suggesting the preference for the mono-exponential ADC measurement. To expedite clinical translation, recommendations towards standardization of DKI and larger studies are needed, similar to that for liver DWI [23].

The main limitation of the present study arises from the small cohort of 20 patients scanned at a single institution. Although we have performed a repeatability study for the DKI/DWI measurements, this was derived from a smaller cohort (5 patients). Nevertheless, one would expect an improvement of the parameters’ repeatability in the context of a larger cohort. In particular, a strong limitation in interpretation of this study’s findings was the large repeatability of K parameter. This could by also improved by changing the acquisition parameters (such as increased number of b values or increased averages) at the expenses of longer scan times.

## Conclusion

In conclusion, our results demonstrate a significant early cohort change to treatment of the diffusion parameter, ADC, in the context of a clinical trial investigating RAS-mutant colorectal liver metastases. In addition, significant non-Gaussian diffusion behaviour (K and D) change was also observed with treatment. The ADC parameter performed better than the K parameter in identifying patients benefitting from the treatment.

## Supplementary Information

Below is the link to the electronic supplementary material.

Supplementary Material 1

## Data Availability

The datasets generated and/or analysed during the current study are not publicly available as appropriate regulatory and institutional approvals are required; datasets might be available from the corresponding author on reasonable request.

## Abbreviations

DWI: Diffusion weighted imaging
ADC: Apparent diffusion coefficient
DKI: Diffusion kurtosis imaging
K: Kurtosis coefficient
D: Kurtosis-corrected apparent diffusion coefficient
DCE-MRI: Dynamic contrast enhancement MRI
WHO: World health organisation
VOI: Volume of interest
r: Spearman correlation coefficient
R%: Repeatability coefficient
RECIST: Response Evaluation Criteria In Solid Tumors
