# Supplementary material for: Apparent diffusion coefficient and kurtosis parameters show early response to anti-angiogenic therapy in patients with colorectal liver metastases
Source: Cancer Imaging. 2026 Jun 4;26:101. doi: 10.1186/s40644-026-01063-3 (PMC13455380; doi:10.1186/s40644-026-01063-3)
Supplement: Supplementary file 1 [file 40644_2026_1063_MOESM1_ESM.docx]

**Supplementary Material**

**Motion Correction of the DKI data**

**In the liver, the dominant source of motion is from breathing, and the bulk of this motion is along the head-foot axis. The DKI was acquired under free breathing, but, with coronal imaging, the lesion displacements between time-points or b-values were largely in plane. An automatic registration procedure was developed, as there was a large number of images per scan needing registration (1200 = 20 slices × 4 repeat averages × 15 diffusion encoding gradients from 5 b-values acquired in 3 orthogonal directions).**

**Motion correction was initiated by manually placing a rectangle over the lesion, ensuring that it encompassed the lesion on all slices. This box was then expanded by 25 pixels, and the computation of the registration cost functions described below were restricted to pixels inside the box. For each registration step, the optimal displacement was determined by exhaustive search of whole pixel shifts in the range +/-20 pixels (head-foot) and +/-10 pixels (right-left). The following 3 procedure steps were applied for each slice.**

1. **For each b-value group:**
   a. **Each image was registered to the b-value group median image using sum of absolute differences (SAD) cost function.**
   b. **The image with the lowest cost was chosen as the target image for the group.**
   c. **All images in the b-value group were registered to this target image using SAD cost function.**
   d. **The trimmed mean image (mean after discarding the lowest and highest values) of the registered images was computed for the b-value group.**
2. **The b=100 s/mm^2^ median image computed in step 1d was used as the target image, to which the median images for the remaining b-values computed in step 1d were registered, using a mutual information cost function.**
3. **All source (unregistered) images were registered to the image with matching b-value resulting from step 2 using a SAD cost function.**
